# Evolutionary Origins of C-Terminal (GPP)n 3-Hydroxyproline Formation in Vertebrate Tendon Collagen

**DOI:** 10.1371/journal.pone.0093467

**Published:** 2014-04-02

**Authors:** David M. Hudson, Rachel Werther, MaryAnn Weis, Jiann-Jiu Wu, David R. Eyre

**Affiliations:** Department of Orthopaedics and Sports Medicine, University of Washington, Seattle, Washington, United States of America; UMR CNRS 5242 - ENS de Lyon- Université Lyon 1, France

## Abstract

Approximately half the proline residues in fibrillar collagen are hydroxylated. The predominant form is 4-hydroxyproline, which helps fold and stabilize the triple helix. A minor form, 3-hydroxyproline, still has no clear function. Using peptide mass spectrometry, we recently revealed several previously unknown molecular sites of 3-hydroxyproline in fibrillar collagen chains. In fibril-forming A-clade collagen chains, four new partially occupied 3-hydroxyproline sites were found (A2, A3, A4 and (GPP)_n_) in addition to the fully occupied A1 site at Pro986. The C-terminal (GPP)_n_ motif has five consecutive GPP triplets in α1(I), four in α2(I) and three in α1(II), all subject to 3-hydroxylation. The evolutionary origins of this substrate sequence were investigated by surveying the pattern of its 3-hydroxyproline occupancy from early chordates through amphibians, birds and mammals. Different tissue sources of type I collagen (tendon, bone and skin) and type II collagen (cartilage and notochord) were examined by mass spectrometry. The (GPP)_n_ domain was found to be a major substrate for 3-hydroxylation only in vertebrate fibrillar collagens. In higher vertebrates (mouse, bovine and human), up to five 3-hydroxyproline residues per (GPP)_n_ motif were found in α1(I) and four in α2(I), with an average of two residues per chain. In vertebrate type I collagen the modification exhibited clear tissue specificity, with 3-hydroxyproline prominent only in tendon. The occupancy also showed developmental changes in Achilles tendon, with increasing 3-hydroxyproline levels with age. The biological significance is unclear but the level of 3-hydroxylation at the (GPP)_n_ site appears to have increased as tendons evolved and shows both tendon type and developmental variations within a species.

## Introduction

Collagens are the main structural component of animal tissues and represent about a third of all proteins in the human body. At least twenty eight types of vertebrate collagen are defined, of which type I collagen is most abundant and perhaps best described [1]. Type I collagen molecules consist of three polypeptide α-chains, approximately 1000 residues in length, each with repeating Gly-Xaa-Yaa primary amino acid sequences folded into the defining triple helical conformation of collagen [2]. Type I collagen is a heterotrimer of two α1 and one α2 chains. Two recent crystallographic studies indicate an α1:α1:α2 registry, with the α2 in the C-terminal trailing position [3], [4]. Type I collagen gene products exhibit clear tissue-specific properties despite having an identical primary sequence in all tissues. Posttranslational and processing variations in collagen chain biosynthesis are a significant source of these structural and functional differences. Indeed, cross-linking chemistry and posttranslational variations are distinct between type I collagens from skin, tendon and bone [5], [6]. Furthermore collagen glycosylation and cross-linking properties can vary within the same tissue during growth and development [7], [8].

Collagen α-chains undergo many posttranslational modifications and processing steps before triple helix formation occurs [9], including prolyl 4-hydroxylation, lysyl hydroxylation and subsequent glycosylation (galactosylhydroxylysine and glucosylgalactosylhydroxylysine) and prolyl 3-hydroxylation. Upon or shortly after secretion from the cell, the N- and C-propeptides are proteolytically removed and telopeptide domain lysine and hydroxylysine residues are converted to aldehydes by lysyl oxidase in preparation for cross-linking and fibril formation.

Interest in the biological significance of collagen posttranslational modifications has increased in the last decade with new insights from the pathobiology of brittle bone disease [10], [11]. Several recessively inherited forms of osteogenesis imperfecta have recently been shown to result from disruptions to collagen posttranslational modifications, processing and trafficking. Notably, significant differences in phenotype are observed with seemingly subtle collagen posttranslational variations. For example, the loss or reduction of triple helical 3-hydroxyproline (3Hyp), triple helical hydroxylysine (Hyl) or telopeptide Hyl can result in osteogenesis imperfecta [12]–[14], Ehlers-Danlos syndrome [15] or Bruck syndrome [16], respectively.

Prolyl 3-hydroxylation is a rare and poorly understood modification that occurs exclusively in collagens. Type I collagen was originally reported to contain a single site of 3Hyp (Pro986 in the α1 chain). The enzyme complex composed of prolyl 3-hydroxylase-1 (P3H1), cartilage associated protein (CRTAP) and cyclophilin B (CypB) was shown to catalyze the 3-hydroxylation of this proline substrate [12], [17]. However, several additional sites have since been reported in A-clade and B-clade collagens [5], [18], many of which exhibit pronounced enzyme and tissue specificity [19], [20]. This is particularly evident in the C-terminal (GPP)_n_ motif of type I collagen, where the 3-hydroxylation is unique to tendon and completely absent in skin and bone [5]. Differential tissue expression of the three members of the P3H family (P3H1, P3H2 and P3H3) probably explains this observation [17], [21], [22]. Indeed, recent results have shown that P3H2 expression in tendon is significantly higher than for P3H1 or P3H3 [19].

To assess the evolutionary origins of the (GPP)_n_ domain as a substrate, we surveyed the pattern of 3Hyp occupancy from early chordates through amphibians, birds and mammals. We examined multiple tissue sources of type I collagen (tendon, bone and skin) and type II collagen (cartilage and notochord) homologs. Type II collagen was included in our study because the known fibrillar collagen genes of pre-vertebrates (lamprey and ciona) have sequence features resembling COL2A1. In the current study evolutionary and tissue-specific variations for prolyl 3-hydroxylation were investigated. The present results support a concept that 3-hydroxyproline residues contribute fundamentally to collagen structure and the diversification of connective tissues.

## Materials and Methods

### Ethics Statement

No human subjects were enlisted for study under this project. All human and animal tissues for this study were obtained from tissue banks and have no identifiers that can link them to an individual living or dead. These tissues are therefore considered minimum risk by the University of Washington and do not require IRB approval. No information has been received by us that could be used to relate specimens to individual living subjects by name or through any other identifier or combination of such. There is therefore no risk either medically or in confidentiality from our acquisition of these tissues or from the data obtained to the subjects or their family members. The laboratory team had no access to patient, surgeon, disease, procedure, date or any other clinical or identification information.

### Collagen extraction

Animal tissues were obtained from various sources. Bovine tissues were obtained from a local abattoir (Crescent Custom Meats, Sumner, WA), chicken was purchased at the supermarket, *Xenopus laevis* was purchased from Xenopus Express, Inc. Mice (C57 black 6) were obtained as a by-product from approved and completed animal studies [12]. Euthanized adult lamprey (*Entosphenus tridentata*) from completed and ongoing animal studies [23] were kindly supplied by Dr. Helena Christiansen, USGS, Western Fisheries Research Center, Columbia River Research Laboratory. Adult human tissue was purchased from the Northwest Tissue Center, Seattle, WA, and fetal human tissue was purchased from an NIH-sponsored institutional tissue bank (Birth Defects Research Laboratory, University of Washington). Type I collagen was solubilized from bone, skin and tendon by heat denaturation for 5 min at 100°C in Laemmli buffer (SDS extraction), 3% acetic acid at 4°C for 24 hours or cyanogen bromide (CNBr) digestion in 70% formic acid at room temperature for 24 hours [18]. Intact type II collagen α-chains were solubilized from cartilage as follows. Tissue slices were digested with chondroitinase ABC prior to extraction with 4M guanidine, 0.05M Tris-HCl pH 7.4 with protease inhibitors for 24 hours at room temperature [18]. Collagen α-chains were resolved by sodium dodecyl sulphate polyacrylamide gel electrophoresis (SDS-PAGE) and stained with Coomassie Blue R-250 (Sigma-Aldrich). Tendon tissue was dissected from the Achilles tendon (human, bovine and mouse), tail (mouse), feet (xenopus and chicken) and mouth (tongue tendon of lamprey).

### Mass spectrometry

Mass spectrometric analysis of 3Hyp content within collagen α-chains was performed as previously described [5], [18]. Collagen α-chains and CNBr peptides were cut from SDS-PAGE gels and subjected to in-gel trypsin digestion. Electrospray mass spectrometry was carried out on the tryptic peptides using an LTQ XL linear quadrapole ion-trap mass spectrometer equipped with in-line Accela 1250 liquid chromatography and automated sample injection (ThermoFisher Scientific). Thermo Xcalibur software and Proteome Discoverer software (ThermoFisher Scientific) were used for peptide identification. Tryptic peptides were also identified manually by calculating the possible MS/MS ions and matching these to the actual MS/MS spectrum. Hydroxyl differences were determined manually by averaging the full scan MS over several minutes to include all the posttranslational variations of a given peptide. Protein sequences used for MS analysis were obtained from the Ensembl genome database.

### Edman N-terminal sequence analysis

Type I collagen was acid-extracted from animal tissues and trypsin digested. The α1(I) and α2(I) chain (GPP)_n_-containing tryptic peptides were separated by reverse-phase HPLC on a C8 column (Brownlee Aquapore RP-300, 4.6 mm×25 cm) with a linear gradient of acetonitrile:n-propyl alcohol (3∶1, v/v) in aqueous 0.1% (v/v) trifluoroacetic acid [5]. 3Hyp content was validated using N-terminal sequence analysis carried out by Edman chemistry on a Porton 2090E machine equipped with on-line HPLC analysis of phenylthiohydantoin derivatives [18].

## Results

### Evolutionary origins

**Figure 1:**
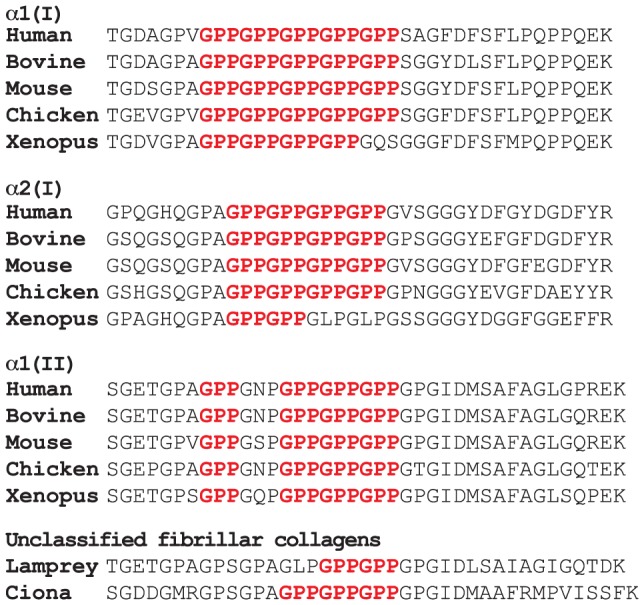
Protein sequence alignment of the collagen (GPP)_n_ motif from phylogenetically diverse animals. Conservation of the (GPP)_n_ motif is shown in red for fibrillar collagens from early chordates through amphibians, birds and mammals. Genomic sequences are from Ensembl. Lamprey and ciona collagen sequences are from *Petromyzon marinus* transcript: COL2A1 ENSPMAT00000009617 and *Ciona intestinalis* transcript: FCOL1 ENSCINT00000014311, respectively.

The A-clade fibrillar collagens contain a repeating sequence of Gly-Pro-Pro residues, which we have coined the (GPP)_n_ motif, located at the C-terminal region of each α-chain in the triple helix [5]. Sequence alignments from the Ensembl database reveal that the (GPP)_n_ motif is highly conserved in fibrillar collagens (Figure 1). According to the Ensembl database, the earliest recognized A-clade fibrillar collagen is in the pre-vertebrate chordate, *Ciona intestinalis* (Figure 1). Indeed, the ciona gene product, predicted from gene *FCOL1* (Ensembl gene ID: ENSCING00000006961), has a (GPP)_n_ motif N-terminal to the C-telopeptide sequence, GPSGPA**GPPGPPGPP**GPGIDMAAFRMPVISSFK. Interestingly, the highly conserved A1 site at Pro986 (GPI**GPP**GPR) is replaced by Ala986 (GPI**GAT**GPR) in *C. intestinalis,* suggesting that the A1 substrate site emerged later in animal evolution.

**Figure 2:**
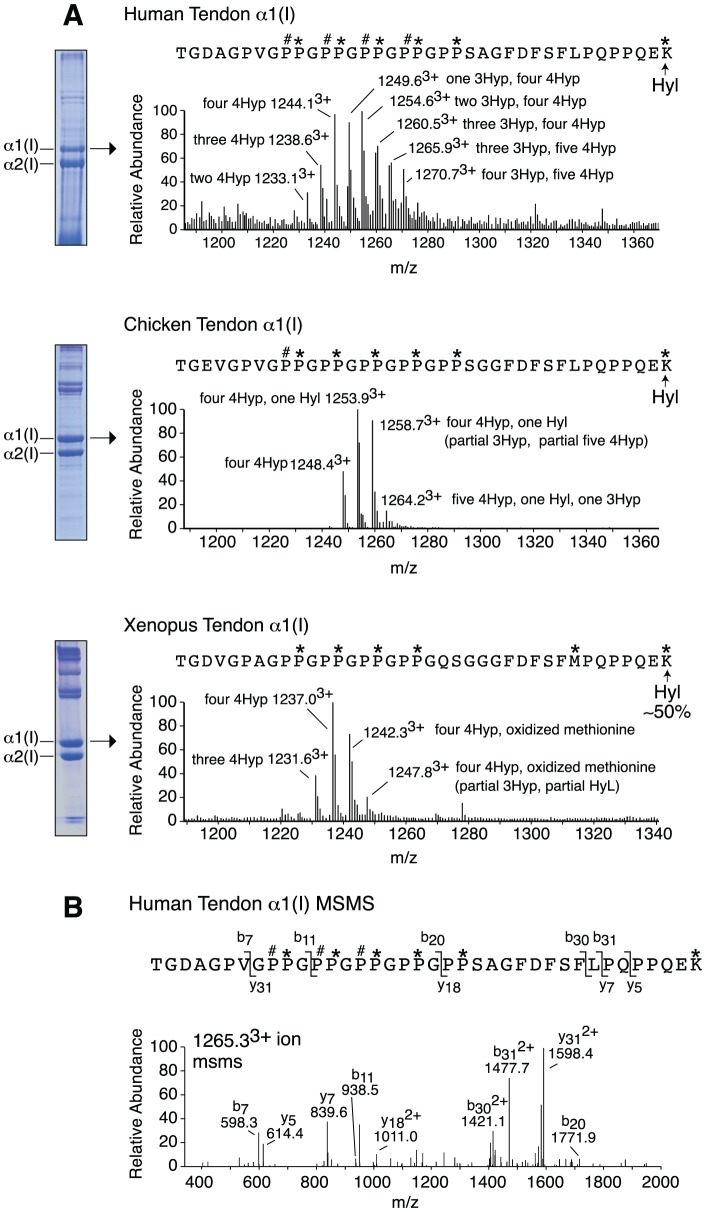
Mass spectra of (GPP)_n_ containing tryptic peptides from adult animal tendons. Full scan spectra from LC-MS profiles of in-gel trypsin digests of α1(I) from human, chicken and xenopus tendon with 6% SDS-PAGE lanes at left (A). MS/MS fragmentation spectrum of the parent ion (1265.3^3+^) from human tendon (B). The sequence is shown with b and y ion breakages. P*, 4Hyp; P#, 3Hyp; K*, Hyl. The cross-linking telopeptide Lys of the human peptide was fully hydroxylated in all posttranslational variants.

**Figure 3:**
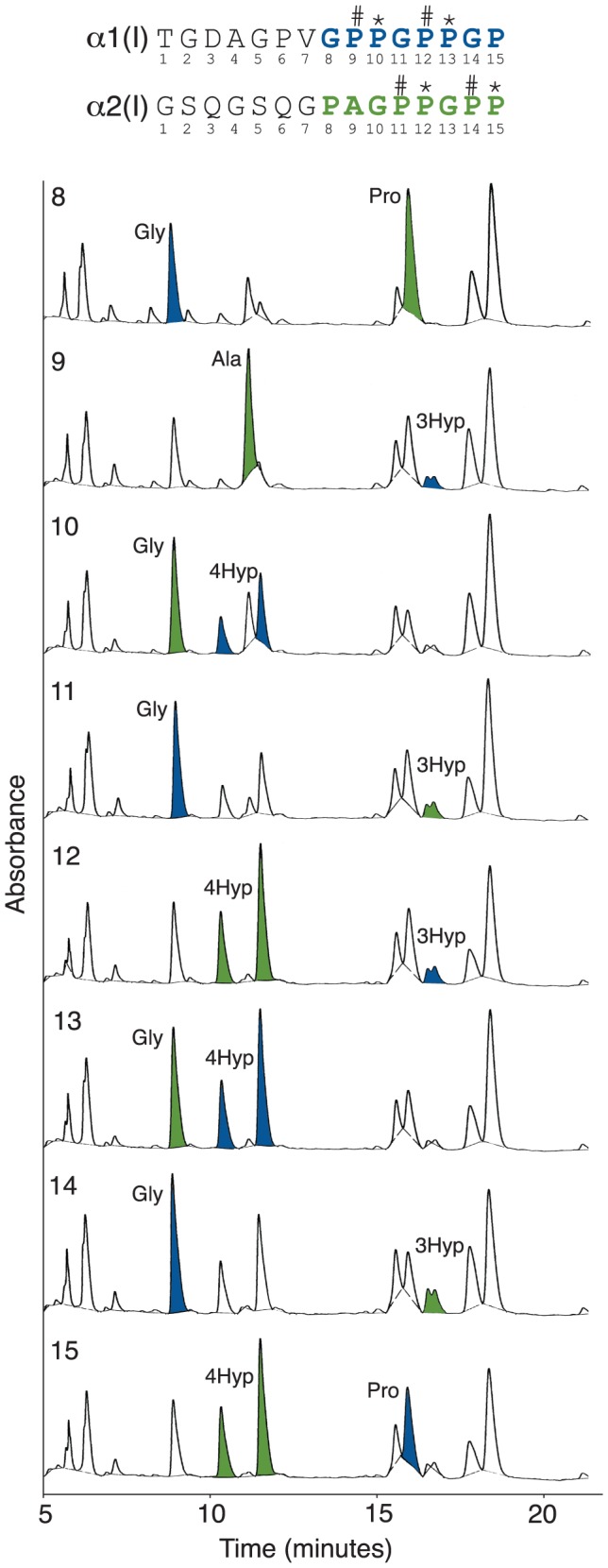
Edman N-terminal sequence analysis confirming 3-hydroxyproline in the (GPP)_n_ motif of tendon type I collagen. The (GPP)_n_-containing tryptic peptides from the α1(I) and α2(I) α-chains of rat tail tendon were recovered as a chromatographic pool and sequenced simultaneously. Sequential phenylthiohydantoin-derivative HPLC chromatograms are shown for sequencer cycles 8-15 (sequencer cycles 1-7 were as predicted from the known sequences of both α-chains). The new residues at each cycle are highlighted in blue for the α1-chain and green for the α2-chain. The 3Hyp residue gave a distinctive double peak as previously reported [18].

We investigated the evolutionary origins of prolyl 3-hydroxylation in the (GPP)_n_ motif of fibril-forming collagens from extant animal connective tissue (bone, skin and tendon) using mass spectrometry. It appears that the (GPP)_n_ motif as a substrate for the modification can be traced to early chordates, as low levels of 3Hyp were found in the α1-chain from lamprey tendon and notochord (Table 1). Further, the (GPP)_n_ seems to have arisen as a substrate for this posttranslational modification in multiple connective tissues. For example, equal levels of 3Hyp were detectable in the (GPP)_n_ from amphibian bone, skin and tendon (Table 1). However, in mammalian type I collagen the (GPP)_n_ motif is a tissue specific substrate site that appears to be acted on predominantly in tendon [5]. It appears therefore that the pronounced tissue-specificity of this substrate for prolyl 3-hydroxylation evolved late in vertebrate evolution. Indeed, birds were evolutionarily the earliest group to reveal significant levels of 3Hyp in tendon, with an average of one residue per (GPP)_n_ in the α2(I) chain. In higher vertebrates (mouse, bovine, human) as many as five 3Hyp residues per (GPP)_n_ motif were found in tendon, with an average of two residues per (GPP)_n_ motif (Figure 2A and B; Table 1). A similar evolutionary or phylogenetic trend was not observed in cartilage α1(II), as low levels of prolyl 3-hydroxylation in the (GPP)_n_ of α1(II) were found in most animals tested (Table 1). The +16 Da mass variants in the MS profiles were verified as 3Hyp by N-terminal Edman sequencing of rat tail tendon type I collagen (Figure 3). The type I collagen (GPP)_n_-containing tryptic peptides from rat and mouse tail tendons have identical amino acid sequences and exhibit analogous MS profiles [5].

**Table 1:** Summary of 3Hyp occupancy in the (GPP)_n_ of type I and II collagen α-chains.

Species	Tendon		Bone		Skin		Cartilage
	α1(I)	α2(I)	α1(I)	α2(I)	α1(I)	α2(I)	α1(II)
Human	1.6 (65%)	2.0 (80%)	0 (0%)	0 (0%)	0 (0%)	0 (0%)	0.3 (30%)
Mouse	1–2 (80%)	1–2 (80%)	0 (0%)	0.3 (30%)	0 (0%)	0.3 (30%)	0.5 (50%)
Bovine	0.2 (20%)	1.2 (50%)	0 (0%)	0.3 (30%)	0 (0%)	0.3 (30%)	0.4 (40%)
Chicken	0.1 (10%)	1.1 (70%)	0 (0%)	0.8 (50%)	0 (0%)	0.7 (50%)	0.1 (10%)
Xenopus	0.1 (10%)	0.1 (10%)	0.1 (10%)	0.1 (10%)	0.1 (10%)	0.1 (10%)	0 (0%)
Species	Tendon		Notochord				
	Unclassified α1		Unclassified α1				
Lamprey	0.15 (15%)		0.05 (5%)				

The table shows the average number of 3Hyp residues per (GPP)_n_ motif with the percentage of α-chains containing at least one 3Hyp residue per (GPP)_n_ given in parentheses. The percentage of each posttranslational variant was determined based on the ratio of the heights of the m/z peaks. For example, the human tendon α1(I) (GPP)_n_ tryptic peptide, TGDAGPV**GPPGPPGPPGPPGPP**SAGFDFSFLPQPPQE**K**, was found to be a mix of eight distinct molecular species giving a hydroxylation (±16 Da) ladder, each representing a posttranslational variant (Figure 2). The molecular location of the each hydroxylated residue (3Hyp, 4Hyp and Hyl) was determined using MS/MS (Figure S1). The C-terminal lysine was predominantly hydroxylated in all Achilles tendons. In this scroll, the 1270.7^3+^ m/z (peptide species containing four 3Hyp residues and five 4Hyp) represents 9% of the total population and the other variations are as follows: 1265.9^3+^ (three 3Hyp residues and five 4Hyp, 10%); 1260.5^3+^ (three 3Hyp residues and four 4Hyp, 13%); 1254.6^3+^ (two 3Hyp residue and four 4Hyp, 19%); 1249.6^3+^ (one 3Hyp residue and four 4Hyp, 16%); 1244.1^3+^ (no 3Hyp residues and four 4Hyp, 18%); 1238.6^3+^ (no 3Hyp residues and three 4Hyp residue, 10%); 1233.1^3+^ (no 3Hyp residues and two 4Hyp residue, 5%). From these percentages, the average number of 3Hyp residues was estimated per α-chain. In this example the calculation is (4×9%)+(3×10%)+(3×13%)+(2×19%)+(1×16%)  =  mean content of 1.6 3Hyp per α1(I) from human tendon. The 3Hyp content in mouse tendon type I collagen was observed to vary markedly with animal age, in the range between one and two 3Hyp residues per (GPP)_n_ as indicated in the table.

### Posttranslational variations between α-chains

**Figure 4:**
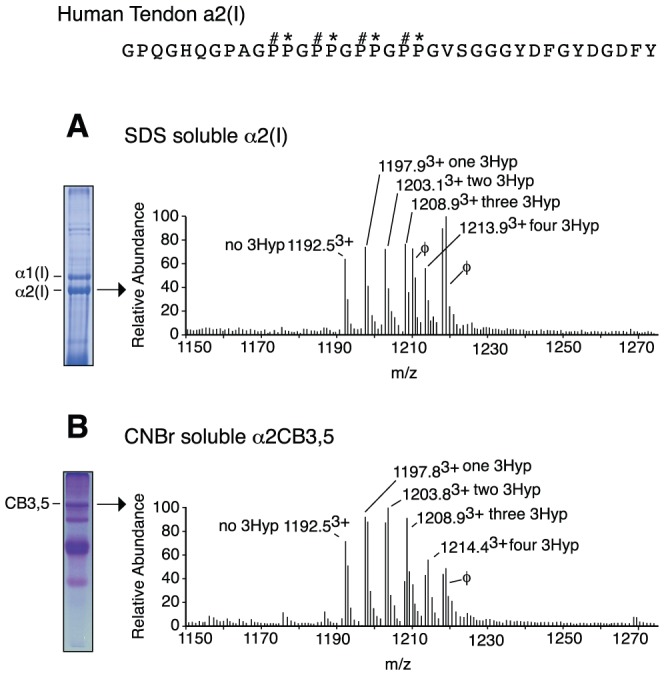
Mass spectra showing prolyl 3-hydroxylation distributed throughout the whole fibril. Collagen was solubilized from adult human tendon using SDS extraction (A) and CNBr digestion (B). Lanes of 6% (A) and 12% (B) SDS-PAGE gels are shown to the left. Similar levels of 3Hyp (∼two 3Hyp per α2(I) chain) were observed using each approach. The 1210^2+^ and 1218^2+^ ions in both ion ladders represent unrelated peptides with a 2+ charge (these ions are indicated with φ).

The α2-chain consistently showed higher levels of prolyl 3-hydroxylation than the α1-chain in all species tested (Table 1). Indeed, while the modification exhibited clear tissue-specificity for tendon in mammals, low levels of 3Hyp were detected in α2(I) of bone and skin from chicken, mouse and bovine (Table 1). The method of collagen extraction was investigated as a potential source of the posttranslational variation observed between α-chains within a fibril. For example, SDS extraction and 3% acetic acid extraction will solubilize only collagen chains and molecules that are not covalently cross-linked in the fibril. In many tendons, particularly mouse and rat tail tendon, acid extracts most of the fibrillar collagen. However, for skin, bone and Achilles tendon from most species, acid-soluble collagen is only 5–20% of the total collagen [24]. Pepsin digestion can solubilize most of the collagen; however this approach gives a heterogeneous population of C-terminal peptides differing in cleavage site (data not shown). This heterogeneity prevented reliable quantification of the posttranslational modification but CNBr digestion could be used to solubilize homogeneous cleavage products. Using this approach, no differences were observed in the levels of prolyl 3-hydroxylation between CNBr digests and SDS extracts of human tendon (both extraction methods yielded peptides and α-chain preparations in which ∼80% had one or more 3Hyps and an overall stoichiometry of approximately two 3Hyp residues per chain). Thus the observed posttranslational modification was similar for cross-linked and non-cross-linked molecules (Figure 4).

### Developmental regulation of prolyl 3-hydroxylation

**Figure 5:**
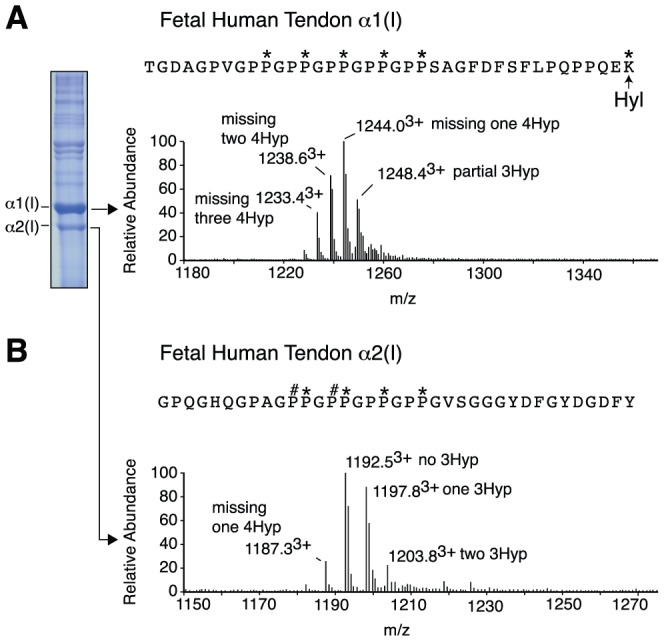
Developmental control of prolyl 3-hydroxylation in tendon. Reduced levels of 3Hyp were observed in fetal tendon relative to adult tissue. MS scan of fetal human Achilles tendon α1(I) with 6% SDS-PAGE lane at left (A). The 1248.4^3+^ ion contains a mix of two peptide posttranslational variants (one 3Hyp and four 4Hyp; and no 3Hyp and five 4Hyp). MS scan of fetal human Achilles tendon α2(I) (B).

Unlike the other substrate sites for prolyl 3-hydroxylation, it is possible that the (GPP)_n_ motif has evolved to be regulated developmentally. For example, the (GPP)_n_-containing peptides had notably high levels of 3Hyp modification when prepared from adult human tendon (Figures 2 and 4). However, from fetal human tendon almost no 3Hyp was detected (<10% occupied with ∼0.1 3Hyp per α1(I) chain; 40% occupied with ∼0.5 3Hyp per α2(I) chain) (Figure 5). In contrast, the α1Pro986 (A1) and α2Pro707 (A3) substrate sites from fetal human tendon are completely 3-hydroxylated (data not shown). Both α1(I) and α2(I) from bovine Achilles tendon displayed a slight increase in (GPP)_n_ 3Hyp occupancy with age (0% 3Hyp in fetal bovine α1(I), 10% in three month calf, 20% in 18 month steer).

An analogous pattern was observed between different tendon types. For example, though high levels of 3Hyp were consistently observed in the tail tendons of mice as in rats [5], the load bearing tendons from the fore limbs and hind limbs revealed even higher levels of 3Hyp (approximately 0.3 to 0.9 residues more 3Hyp per α-chain on average). Interestingly, both the developmental and tendon type variations were more pronounced for the α1-chain than the α2-chain. The latter tended to have constitutively higher 3Hyp occupancy in its C-terminal (GPP)_n_ motif.

## Discussion

Prolyl 3-hydroxylation is a highly conserved collagen posttranslational modification found throughout the animal kingdom [18], [24]. The evolutionary origins of this ancient modification can be traced as far back as porifera, the most primitive extant multicellular animal [25]. Genomic duplications in early chordates gave rise to the three P3H isoenzymes (P3H1, P3H2 and P3H3) encoded in higher vertebrate genomes [26]. P3H2 is the predicted modifying enzyme for type IV collagen substrates in the basement membrane [22], suggesting that P3H2 may be the most conserved in substrate specificity of the duplicated and evolving isoenzymes. It has been demonstrated using cell line RNA interference that the (GPP)_n_ motif of collagen types I and II is a substrate modified by P3H2 [27]. The (GPP)_n_ motif of fibrillar collagens therefore appears to have evolved as a potential substrate for a pre-existing modifying enzyme that is used to hydroxylate such sequences in type IV collagen. The degree of prolyl 3-hydroxylation may be dependent on the tissue expression patterns of the enzyme [19].

The (GPP)_n_ 3Hyp appears to have arisen prior to early vertebrate evolution, as low levels of the modification were detected in lamprey tissues. Low levels of 3Hyp were also found equally distributed across type I collagen from xenopus bone, skin and tendon. Nevertheless, in screening the (GPP)_n_ motif of type I collagens prepared from different major tissue types, significant 3Hyp occupancy was unique to tendon. Tissue specificity is first observed in chicken, where the 3-hydroxylation is more prevalent in tendon type I collagen. In mammals, 3-hydroxylation of the (GPP)_n_ in type I collagen appears to have become exclusively regulated in tendon.

Tendons first evolved as sheet-like structures that transmitted muscle force over a wide area, such as myosepta in Cephalochordates [28]. Evolutionarily, the first occurrence of tendons as linear, dense, fibrous structures capable of transmitting muscle force appears in the Agnathans [28]. Interestingly, these tendons, which control the protrusion and retraction mechanism of the tongue, evolved before the advent of vertebrates or endochondral bone. Cartilaginous and bony fish also have linear tendons with similar mechanical properties to mammals, but with broadly different fascicle organization [29]. The type I collagen (GPP)_n_ motif appears to lack significant levels of 3Hyp in the tissues of bony fish (Hudson and Eyre, unpublished observation). In mammals, tendon is composed predominantly of collagen that is organized into fibrils, fibers, fiber bundles and fascicles. Type I collagen from tendon has several distinctive properties from that of bone and skin, including its material properties, manner of cellular assembly, cross-linking chemistry and posttranslational variations [30], [31].

Developmentally in human tendon it is clear that prolyl 3-hydroxylation of the (GPP)_n_ substrate is highest in the adult. Indeed, fetal human tendon contained almost no (GPP)_n_ 3Hyp in α1(I) and much less in α2(I). This finding may provide insight into the specificity of the modifying isoenzyme (most likely P3H2) and suggests that 3Hyp has a functional role in tendon development. Tendon fibrils are known to increase in diameter as a function of age, a phenomenon that can also affect the tissues' material properties [32]. Developmental changes in mammalian tendon such as increased stiffness, resilience and elastic storage capability are commonly attributed to increased or altered cross-linking during tendon growth and maturation [7], [33]. For example, stable covalent cross-links have previously been shown to increase with age in bovine Achilles tendon collagen [7]. Potential effects from increases in other posttranslational modifications have received less attention. The (GPP)_n_ prolyl 3-hydroxylation substrate is an interesting candidate for further study as it appears to be developmentally regulated and peculiar to tendon. This suggests that as the fibrils grow in diameter by accretion of new collagen molecules [30], there may be changes in the 3Hyp content and potentially related posttranslational modifications that can accommodate growth while maintaining tissue strength and integrity.

The preferential modification of α2(I) (GPP)_n_ over α1(I) may have functional implications. We have previously proposed a role for 3Hyp in the supramolecular assembly of adjacent collagen triple helices through intermolecular hydrogen bonding [34]. The D-spacing between certain 3Hyp residues in the α-chain also suggests a role in fine-tuning the D-periodic relationship between molecules, which is necessary for mature cross-linking. It is possible that the C-terminal 3-hydroxyls in α2(I) could aid in forming intramolecular interactions through water-mediated hydrogen bonds or electrostatic interactions, similar to what has been suggested for the A1 3Hyp site in α1(I) [34]. Such interactions could direct the chain registry during triple helix initiation of heterotrimeric type I collagen. The chain registry of type I collagen has not been established for any tissue with any certainty, but 3Hyp in the initiating C-terminal (GPP)_n_ repeat could be a specific regulator for tendon.

Defining the evolutionary origins of the (GPP)_n_ 3Hyp is potentially important for understanding the functional significance of prolyl 3-hydroxylation in general. We assume that hydroxylation of this 3Hyp substrate is regulated during synthesis in the ER and plays a role in molecular folding, fibril assembly and the unique properties of the resulting fibrillar architecture of tendon. Posttranslational modifications, particularly cross-linking, are believed to be important in modulating the tissue-specific properties of type I collagens. The present findings support a concept that the 3Hyp collagen modification developed its substrate specificity during vertebrate evolution and contributed fundamentally to collagen fibril structure and the diversification of connective tissues.

## Supporting Information

Figure S1 **MS/MS fragmentation patterns from each identified post-translational variant ion peak of the (GPP)_n_ motif from human tendon α1(I) collagen.** The posttranslational variant and its MS/MS fragmentation spectrum are shown for each parent ion in the hydroxylation ladder (±16 Da) from figure 2A. The sequence is shown with b and y ion breakages. P*, 4Hyp; P#, 3Hyp; K*, Hyl. The cross-linking telopeptide Lys of the human peptide was found to be essentially fully hydroxylated in all posttranslational variants.
(TIF)
