# Supplementary figures and images for: Evolutionary Origins of C-Terminal (GPP)n 3-Hydroxyproline Formation in Vertebrate Tendon Collagen
Source: PLoS One. 2014 Apr 2;9(4):e93467. doi: 10.1371/journal.pone.0093467 (PMC3973637; doi:10.1371/journal.pone.0093467)

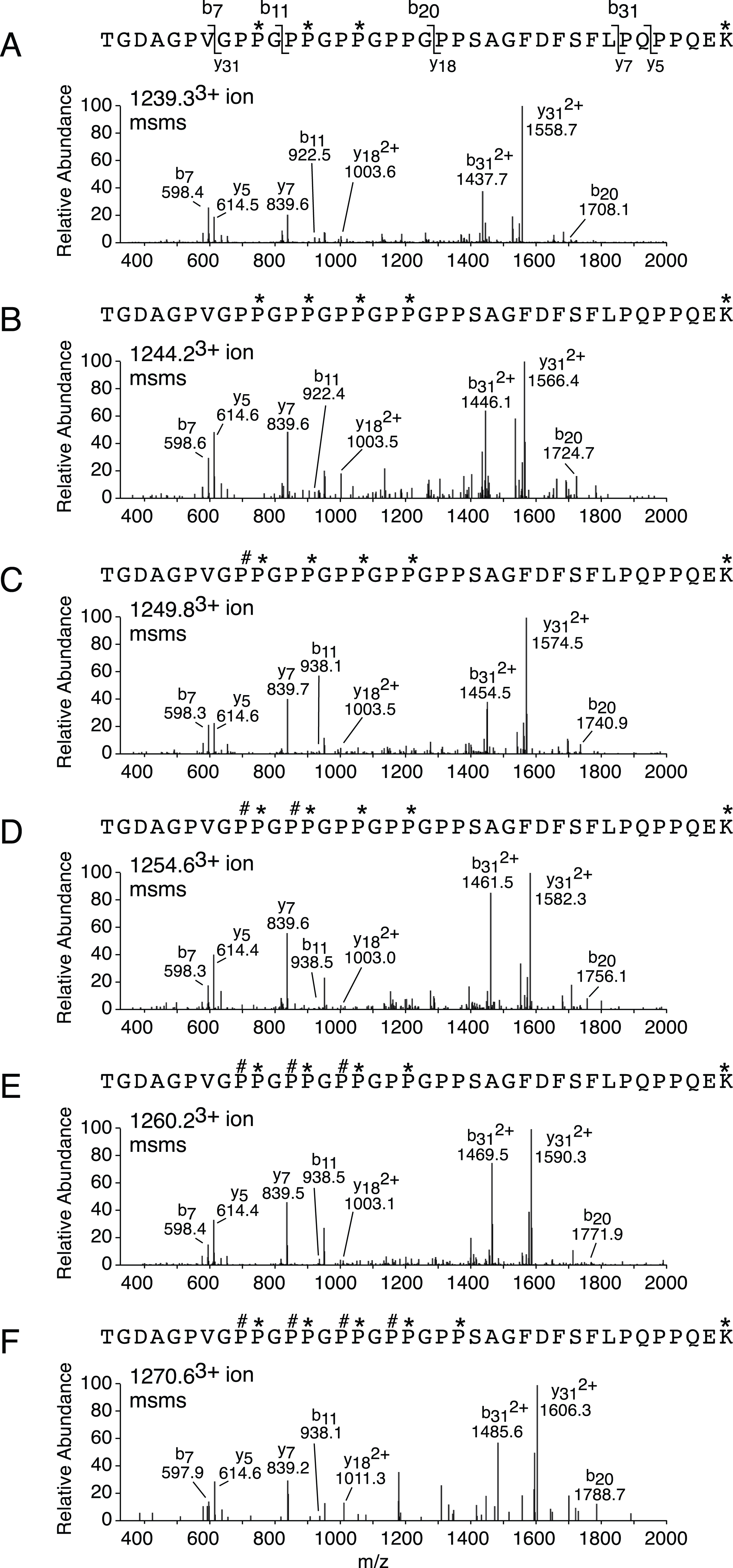

Supplement: Figure S1 — MS/MS fragmentation patterns from each identified post-translational variant ion peak of the (GPP)n motif from human tendon α1(I) collagen. The posttranslational variant and its MS/MS fragmentation spectrum are shown for each parent ion in the hydroxylation ladder (±16 Da) from figure 2A. The sequence is shown with b and y ion breakages. P*, 4Hyp; P#, 3Hyp; K*, Hyl. The cross-linking telopeptide Lys of the human peptide was found to be essentially fully hydroxylated in all posttranslational variants. (TIF) [file pone.0093467.s001.tif]
